# THE UNIVERSITY OF OKLAHOMA GSA STUDENT CHAPTER AND MULTICAMPUS COORDINATION AND COLLABORATION

**DOI:** 10.1093/geroni/igad104.0604

**Published:** 2023-12-21

**Authors:** Keith Kleszynski

**Affiliations:** University of Oklahoma Health Sciences Center, Oklahoma City, Oklahoma, United States

## Abstract

The University of Oklahoma is a public university system comprised of a main campus in Norman, Oklahoma, a health sciences campus in Oklahoma City, a campus in Tulsa, Oklahoma, as well as robust online education programming. The University of Oklahoma GSA Student Chapter launched in late 2022 and is open to student membership from all campuses within the University of Oklahoma system. Student membership recruitment is facilitated by a team of inter-professional faculty whom seek to engage any learner on any campus with an interested in gerontology in its broadest sense. The University of Oklahoma GSA Student Chapter utilizes an umbrella and sub-committee structure to maintain governance. This structure is a result of inter-disciplinary collaboration across campuses, colleges, and among faculty and is replicable in university settings with multiple campuses and diverse sets of learners.

